# Four new species of *Cyrtandra* (Gesneriaceae) from the South Pacific islands of Fiji

**DOI:** 10.3897/phytokeys.91.21623

**Published:** 2017-12-15

**Authors:** Melissa A. Johnson

**Affiliations:** 1 Rancho Santa Ana Botanic Garden, Claremont Graduate University, 1500 N. College Ave., Claremont, CA 91711, USA

**Keywords:** *Cyrtandra*, Gesneriaceae, Fiji, South Pacific, islands, new species, taxonomy, conservation

## Abstract

During fieldwork in Fiji, four new species of *Cyrtandra* (Gesneriaceae) were discovered and are described herein: *C. gregoryi* M.A.Johnson, **sp. nov.**, *C. hispida* M.A.Johnson, **sp. nov.**, *C. longifructosa* M.A.Johnson, **sp. nov.**, and *C. waisaliensis* M.A.Johnson, **sp. nov.** The addition of four new species brings the current number of Fijian *Cyrtandra* to 41 endemic species. Two of the four species are known from only a single locality, and all of the new species are likely endangered or critically endangered. Continued fieldwork in the islands of Fiji is warranted in order to better understand current species distributions and population demographics of *Cyrtandra* in this species-rich and still poorly explored region of the South Pacific.

## Introduction

The Southeast Asian-Pacific genus *Cyrtandra* J.R. Forster & G. Forster (Gesneriaceae) comprises ca. 800 species, with centers of diversity in Borneo, New Guinea, the Philippines, and the Pacific islands (Atkins et al. 2013). Species of *Cyrtandra* are restricted to the understory of rainforests and exhibit high diversity in habit (shrubs, small trees, herbs, or vines), flower color (white, yellow, purple, pink, red), and fruit morphology (indehiscent capsules or berries). In the Pacific, *Cyrtandra* is one of the largest and most widely distributed genera of flowering plants, with ca. 175 species occurring across a region that extends from the Solomon Islands, east to the Marquesas, and north to the Hawaiian Islands. The vast majority of species are single island endemics, with the entire range of a species often being restricted to a single valley or mountain region.

Recent phylogenetic studies suggest that *Cyrtandra* evolved in Southeast Asia, followed by dispersal to the Pacific islands (Clark et al. 2008, 2009; Johnson et al. 2017), likely via frugivorous birds. The Pacific clade appears to have originated in Fiji ca. 9 mya, with subsequent founder events from Fiji to archipelagos both near (e.g., Samoa) and far (e.g., the Hawaiian Islands) resulting in the current distribution of *Cyrtandra* across the Pacific (Johnson et al. 2017). While Fiji hosts the second highest number of endemic *Cyrtandra* species in the Pacific (second only to the Hawaiian Islands, with 60 spp.), only limited research has been conducted on the genus in this diverse region. Gillett (1967) conducted the only thorough taxonomic review of Fijian *Cyrtandra* to date, with his treatment dividing 35 species among six informal groups. However, upon further study of *Cyrtandra* across the South Pacific, Gillett (1973) acknowledged that these groupings were largely inadequate and that a more accurate treatment of Fijian *Cyrtandra* would require considerably more fieldwork. Smith’s treatment of *Cyrtandra* in the Flora of Fiji (1991) was largely drawn from Gillett (1967), although two species were restored from synonymy bringing the number of recognized species to 37.

Botanical explorations in Fiji from 1840–1953 resulted in the description of all 37 currently recognized species of Fijian *Cyrtandra*. Since the 1960s, relatively few collections of Fijian *Cyrtandra* have been made. In the 1970s, a number of new roads were built in Fiji to accommodate the expanding agricultural industry, vastly increasing accessibility into remote regions (e.g., central Vanua Levu, eastern Taveuni; Lin 2012). In 2014 and 2015, I was able to undertake extensive fieldwork across the four largest Fijian islands (Viti Levu, Vanua Levu, Taveuni, and Kadavu), focusing on regions that have been poorly explored in the past. This work resulted in the discovery of four new species that are here described and illustrated.

## Methods

Diagnoses of the new species are based on morphological traits and DNA sequence variation in a phylogenetic context. Morphological measurements were taken from live plants in the field, as many characters essential to *Cyrtandra* identification are lost upon drying (particularly floral characters). Information was also taken from liquid fixative-preserved flowers and fruit, as well as from digital photographs. To ensure accurate identification, comparisons were made with all existing species descriptions (Gillett 1967, Smith 1991) as well as with herbarium specimens housed at BISH, GH, K, NY, PTBG, RSA, SUVA, UC, and US. Samples of all four new species were included in a recent molecular phylogeny of the Pacific clade of *Cyrtandra*, which is based on five loci and a dense taxon sampling of 121 species (including 30 Fijian species; Johnson et al. 2017). This study provided the information necessary to identify the closest relatives of each of the new species based on shared phylogenetic history. Conservation status was assessed in accordance with IUCN Red List Category criteria (IUCN Standards and Petitions Subcommittee 2016).

## Taxonomic treatment

### Cyrtandra gregoryi

Taxon classificationPlantaeLamialesGesneriaceae

M.A.Johnson sp. nov.

urn:lsid:ipni.org:names:60475584-2

Figs 1 , 2

#### Diagnosis.

*Cyrtandra gregoryi* is closely related to *C. ciliata* Seem. (Fig. 2), but differs in its elliptic to ovate leaves up to 39 × 17 cm (vs. lanceolate to ovate leaves up to 28 × 12 cm), axillary inflorescences with indument of brown trichomes (vs. cauliflorous inflorescences with indument of white trichomes), pale green calyces 6–13 mm long that are cleft unequally into lanceolate coriaceous lobes (vs. calyces white, 7–10 mm long, cleft nearly to the base into equal linear-lanceolate lobes), these splitting along two to three sutures and recurving after anthesis (vs. remaining erect after anthesis), and corollas with exserted style and stamens (vs. style and stamens included).

#### Type.

FIJI. Taveuni: near the end of the Lavena coastal walk along the Wainibau stream, ca. 3.5 km NW of Lavena Village, 16°52.10'S, 179°54.32'W, 32 m elev., 04 August 2014, *M.A. Johnson 105* with G.J. Hora (holotype: RSA).

**Figure 1.**
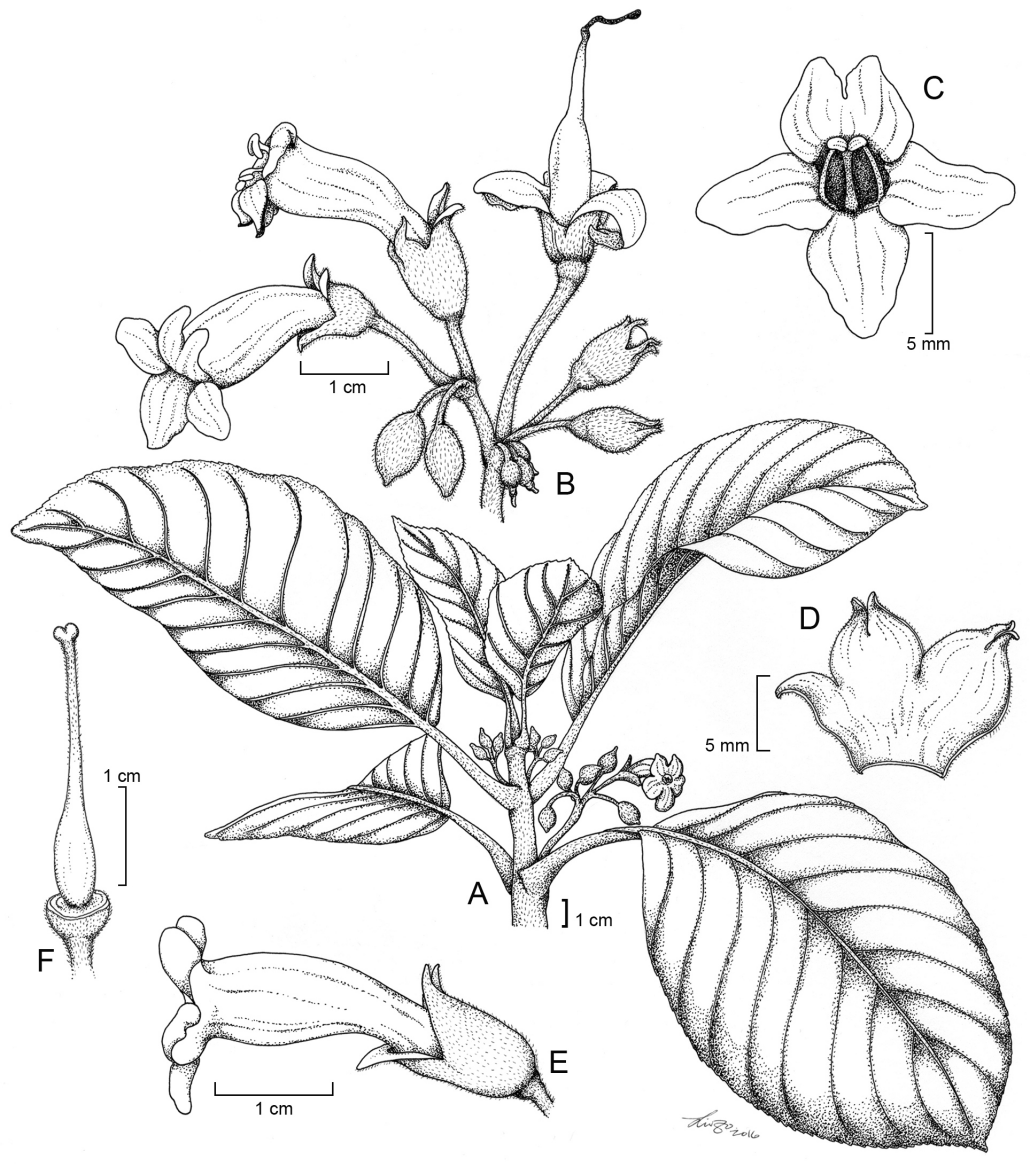
*Cyrtandra gregoryi* M.A.Johnson **A** Habit **B** Inflorescence **C** Corolla, anterior view **D** Calyx **E** Flower, lateral view **F** Gynoecium. Drawn from Johnson 105 (RSA) and field images.

**Figure 2.**
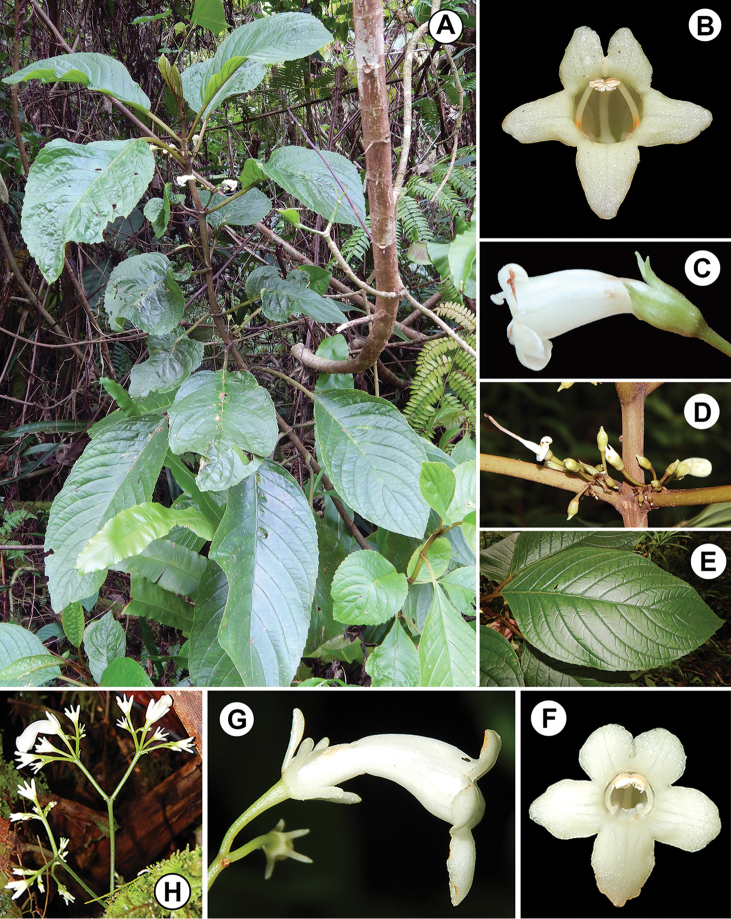
*Cyrtandra gregoryi* M.A.Johnson and closest relative (*C. ciliata*) based on a molecular phylogeny by Johnson et al. (2017). **A** *C. gregoryi* shrub habit **B** *C. gregoryi* corolla, anterior view **C** *C. gregoryi* flower, lateral view **D** *C. gregoryi* axillary cyme inflorescence **E** *C. gregoryi* adaxial leaf surface **F** *C. ciliata* corolla, anterior view **G** *C. ciliata* flower, lateral view **H** *C. ciliata* cauliflorous cyme inflorescence. All photos taken in the field by M. Johnson.

#### Description.

Shrub 0.9–2.2 m tall; *stems* unbranched to few-branched, with a dense indument of dark brown uniseriate multicellular trichomes ca. 0.5 mm long. *Leaves* opposite, internodes 2–7 cm long, blades elliptic to ovate to obovate, 22–39 cm long, 7–17 cm wide, upper surface sparsely strigillose, lower surface glabrate except for the densely pubescent 9–13 secondary veins on each side, margins serrulate to subentire, apex acute, base cuneate to rounded, petioles 5–11 cm long, densely pubescent with short brown trichomes; *inflorescence* an axillary cyme with dense brown pubescence on the peduncles and pedicels, 3–22 flowers, cymules 1–4 flowered, peduncle to 31 mm long, terminated by bracts to 6 mm long, narrowly lanceolate, deciduous after anthesis, pedicels to 31 mm long; *calyx* 6–13 mm long, outer surface pale green and moderately pubescent with appressed dark brown uniseriate trichomes, inner surface white and papillate, unequally cleft into 5 lanceolate coriaceous lobes, 3–10 mm long, apex acuminate, upper lobes occasionally connate, often continuing to split along two or three sutures and curl back after anthesis, deciduous; *corolla* white, tube funnelform, slightly curved near mid point, 23–27 mm long, 6–9 mm wide, outer surface glabrate to densely pubescent with short glandular trichomes, inner surface glabrous, upper lobes 5–7 mm long and 5–6 mm wide, lower lobe 7–8 mm long and 5–6 mm wide, lobes recurving after anthesis; *stamens* 2, ca. 9 mm long, becoming exserted from the corolla tube during ovulate phase, base of the filaments bright orange, anthers apically connate, staminodes 3; *nectary disc* cupulate, annular; *gynoecium* (ovary, style, and stigma) 16–24 mm long, ovary glabrous, style pubescent with capitate glandular trichomes, exserted, stigma shallowly bilobed; *fruit* not seen.

#### Distribution and ecology.

**Figure 3.**
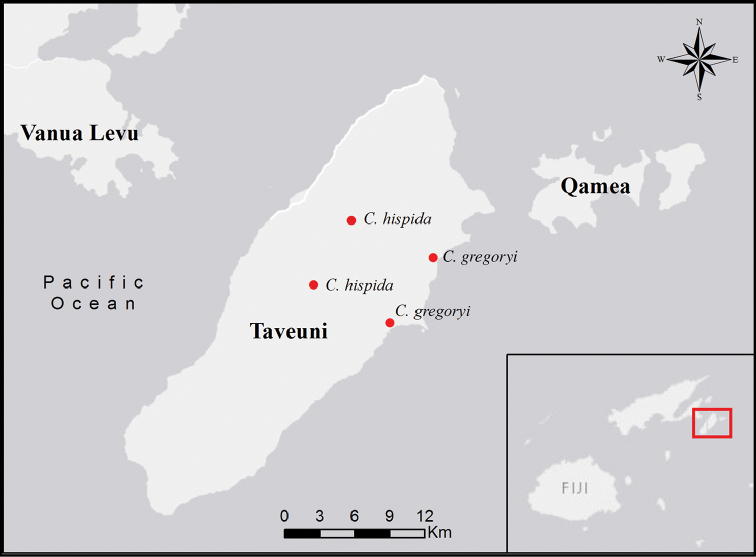
Distribution of *Cyrtandra gregoryi* and *C. hispida* on Taveuni, Fiji.

*Cyrtandra gregoryi* is only known from two locations in eastern Taveuni, Fiji, where it grows in lowland forests and along stream banks from ca. 30–50 m (Fig. 3).

#### Phenology.

Individuals of this species were in flower when collected in August and November, with fruits likely becoming mature ca. 5–6 months later.

#### Etymology.

I am pleased to name this new species after my husband, Gregory Hora, to whom I am most grateful for his assistance in collecting this and other species across Fiji.

#### Phylogenetic placement.

**Figure 4.**
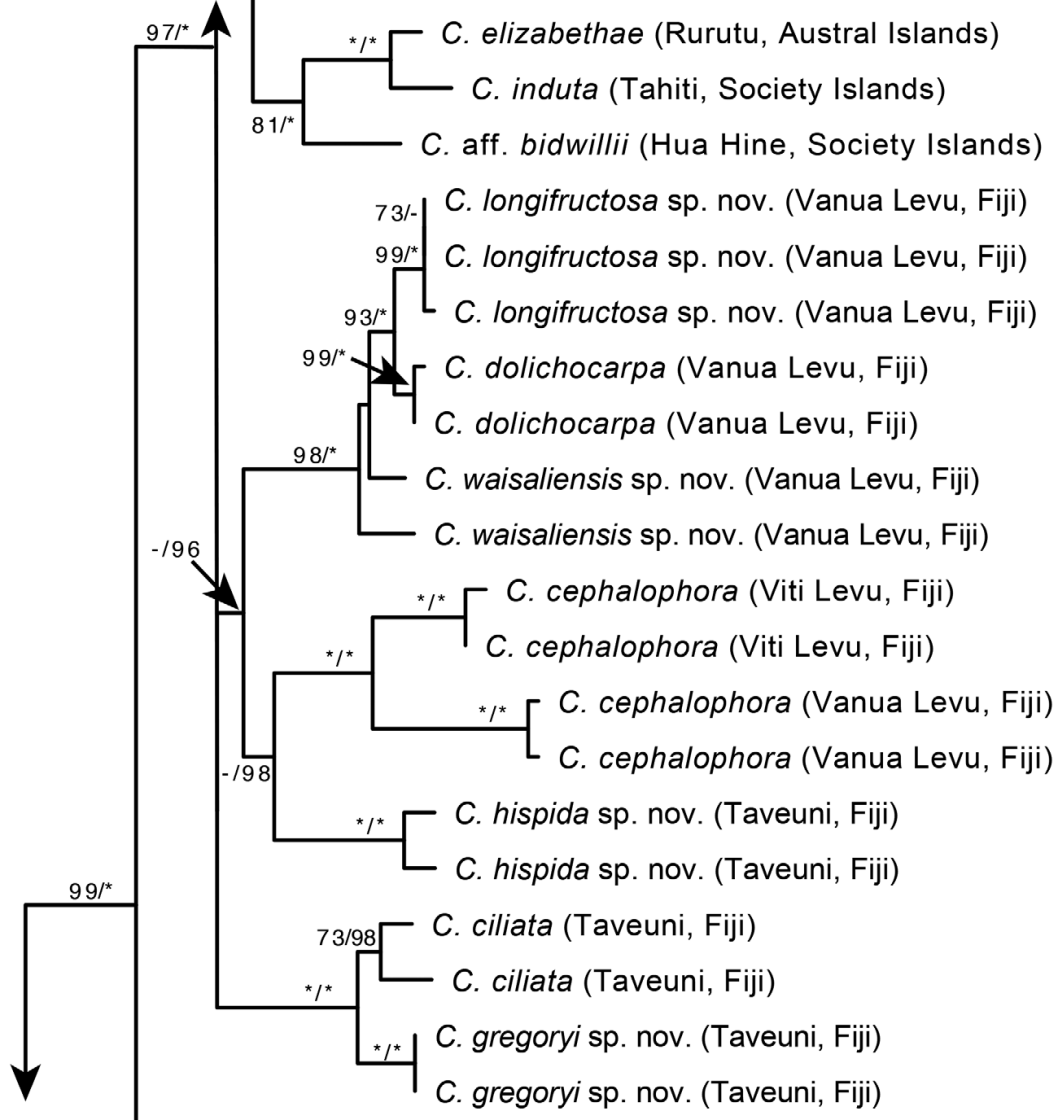
A portion of the Maximum Likelihood phylogram from Johnson et al. (2017) based on three nuclear (ITS, ETS, *Cyrt*1) and two chloroplast (*psb*A-*trn*H, *rpl*32-*trn*L) loci. Support values shown for each branch are bootstrap and posterior probabilities when ≥ 50% and ≥ 0.50, respectively. An asterisk indicates 100% BS or 1.0 PP; a dash indicates that the branch was not supported.

A recent phylogenetic study by Johnson et al. (2017) placed *Cyrtandra gregoryi* sister to *C. ciliata* with strong support (Fig. 4). *Cyrtandra ciliata* is endemic to the Fijian islands of Vanua Levu, Taveuni, and Koro from 300–1100 m elevation. These species share a cymose inflorescence and glabrous to glabrate leaves. The key provided in the taxonomic treatment by Gillett (1967) would place *C. gregoryi* in species Group 5 based on the branching cyme inflorescence, non-woody inflorescence axis, deciduous calyx, styles and/or stamens being exserted from the corolla tube, and stamens 8–12 mm long.

#### Conservation status.

Proposed IUCN Red List Category: Endangered (EN) based on an estimated area of occupancy of < 500 km^2^ (criterion B2), known to exist at no more than five locations (B2a), inferred decline in area of occupancy (B2bii), decline in area, extent, and/or quality of habitat (B2biii), decline in number of mature individuals (B2bv), and population size estimated to number fewer than 250 mature individuals (D).

Although Bouma National Heritage Park protects ca. 15,000 hectares of intact rainforest on eastern Taveuni, indigenous Fijians are permitted to clear land near villages for agriculture. As a result, large areas of coastal forest are increasingly being cleared for dalo (taro, *Colocasia esculenta* (L.) Schott) and yaqona (kava, *Piper methysticum* L.f.), the two main export crops of Taveuni. Given that *C. gregoryi* appears to be restricted to low-elevation forests, it is highly likely that individuals of this species were extirpated during clearing for human settlements and agriculture. Invasive plants are also a major threat to native plants in the area; mile-a-minute vine (kudzu, *Pueraria lobata* (Willd.) Ohwi) may be particularly problematic as it rapidly grows over trees and shrubs and can kill other plants with heavy shading. Lastly, anthropogenic-induced climate change is a threat to island plant communities. Tropical cyclones are expected to increase in intensity and severity in the coming years (Emmanuel 2005, Knutson 2010), and can have devastating effects on island vegetation due to high winds, flooding, and storm surges (e.g., Cyclone Pam in 2015 caused extensive damage to Vanuatu’s forests). Most recently, Cyclone Winston, the strongest tropical cyclone to make landfall in the South Pacific Basin in recorded history, ravaged the islands of Fiji in February of 2016. The coastal regions of eastern Taveuni were inundated by massive storm surges, and much of the vegetation was damaged by winds of up to 185 mph. Although forested regions can often regenerate after a natural disaster if given sufficient time, recent research in the South Pacific suggests that a cyclone can be a catalyst for human-coping strategies that increase pressure on forest ecosystems and exposes them to invasive plant species (Goulding et al. 2016).

#### Additional specimens examined.

FIJI. Taveuni: Tavoro Falls Trail in Bouma National Heritage Park, 19 November 2016, *J.C. Game 16/235* with S. Fawcett (PTBG).

#### Notes.

Eight individuals of *C. gregoryi* were recorded during field surveys along the Lavena coastal trail, with all of these being reproductive. Additional field surveys in the area are likely to reveal more individuals. No other *Cyrtandra* species were observed growing sympatrically with *C. gregoryi* in the Lavena region, although *C. tempestii* Horne ex. C.B. Clarke was collected 0.64 km to the SE. An additional collection was made of a single individual of *C. gregoryi* near the Tavoro Falls in Bouma National Heritage Park, an area that also hosts *C. ciliata*.

### Cyrtandra hispida

Taxon classificationPlantaeLamialesGesneriaceae

M.A.Johnson sp. nov.

urn:lsid:ipni.org:names:77174061-1

Figs 5 , 6

#### Diagnosis.

*Cyrtandra hispida* is morphologically similar to C. waisaliensis sp. nov., but differs in its axillary cyme inflorescence of 2–4 flowers (vs. cauliflorous cyme inflorescence of 2–8 flowers), green bracts and bracteoles 5–9 mm long (vs. white bracts and bracteoles 3–10 mm long), calyx pale green and 29–31 mm long (vs. calyx white and 23–37 mm long), corolla tube 31–34 mm long (vs. corolla tube 23–27 mm long), and staminodes 2 (vs. staminodes 3).

#### Type.

FIJI. Taveuni: Des Voeux Peak, 16°50.48'S, 179°57.97'W, 1109 m elev., 13 July 2014, *M.A. Johnson 91* with G.J. Hora (holotype: RSA; isotype: SUVA).

**Figure 5.**
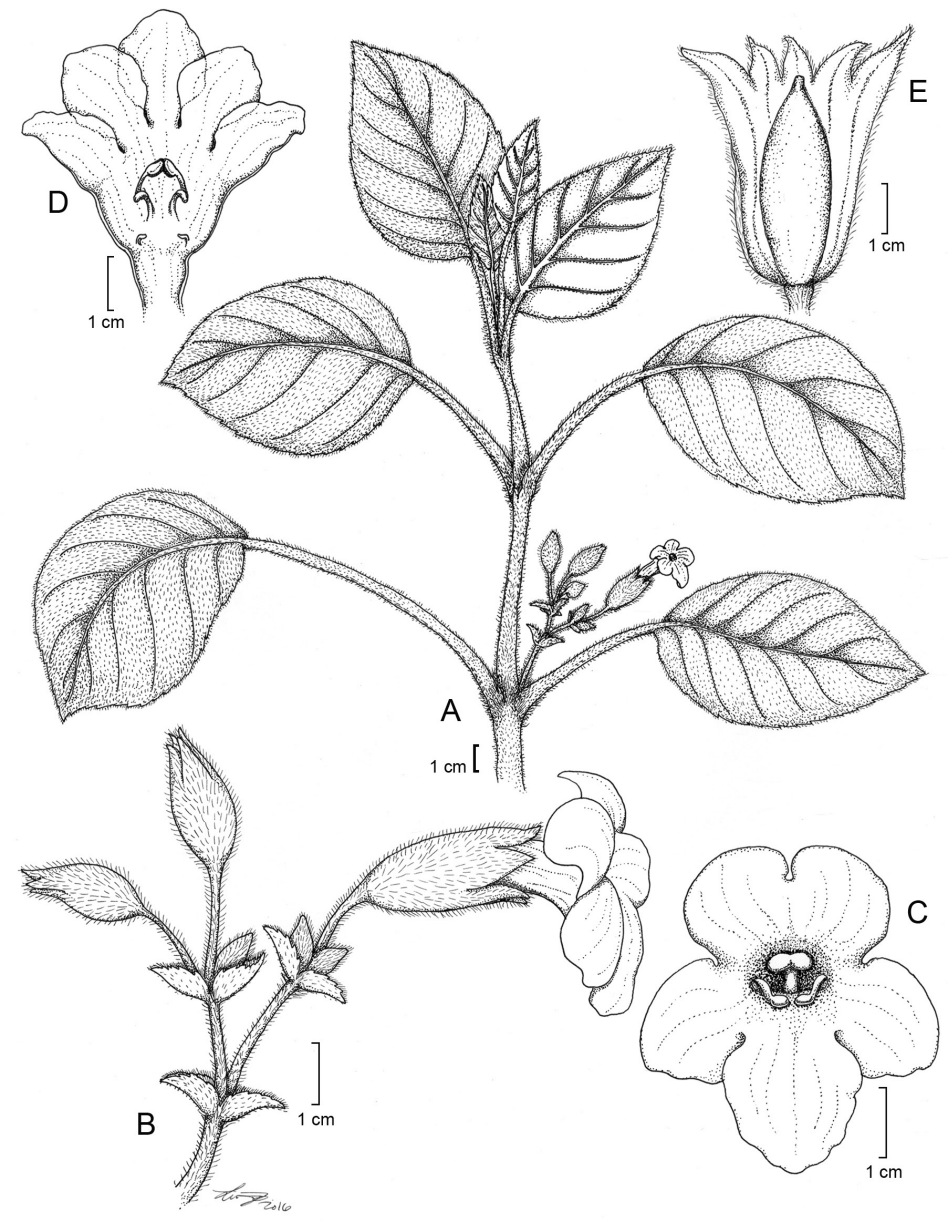
*Cyrtandra hispida* M.A.Johnson. **A** Habit **B** Inflorescence **C** Corolla, anterior view **D** Corolla, longitudinal section **E** Calyx, longitudinal section and young fruit. Drawn from Johnson 91 (RSA, SUVA), Johnson 212 (SUVA), Johnson 215 (RSA), and field images.

**Figure 6.**
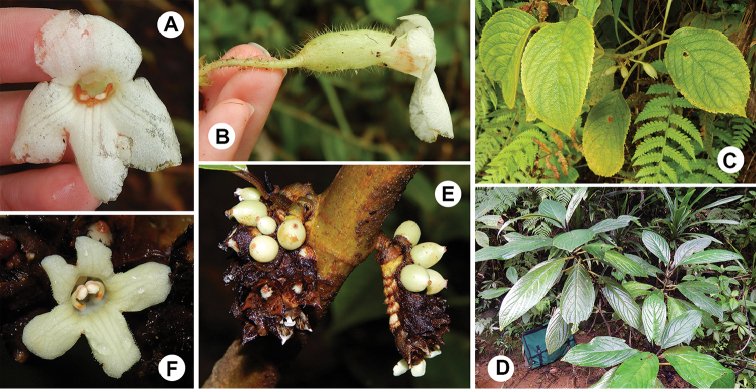
*Cyrtandra hispida* M.A.Johnson and closest relative (*C. cephalophora*) based on molecular phylogeny by Johnson et al. (2017). **A** *C. hispida* corolla, anterior view **B** *C. hispida* flower, lateral view **C** *C. hispida* shrub habit and axillary cyme inflorescence **D** *C. cephalophora* shrub habit **E** *C. cephalophora* capitate-cylindrical cauliflorous inflorescence and young fruits **F** *C. cephalophora* corolla, anterior view. All photos taken in the field by M. Johnson, with photos of *C. hispida* taken from the type collection.

#### Description.

Shrub 0.6–1.2 m tall; *stems* unbranched or few-branched, with light brown hispid uniseriate multicellular trichomes ca. 5 mm long. *Leaves* opposite, internodes 3–9 cm long, the blades oval to ovate to obovate, 15–22 cm long, 8–10 cm wide, upper and lower surfaces densely strigose with light brown uniseriate trichomes to 2 mm long, 5–7 secondary veins on each side, margins serrate to biserrate, apex acute to acuminate, base oblique or aequilateral and rounded to cuneate, petioles 4–9 cm long, densely pubescent with hispid trichomes ca. 6 mm long; *inflorescence* an axillary cyme, 2–4 flowered, densely hispid throughout, peduncle 10–14 mm long, terminated by green bracts 5–9 mm long, ovate to narrowly lanceolate, densely hispid, pedicels 27–28 mm long, often subtended by bracteoles similar to the outer bracts; *calyx* pale green, cylindrical, 29–31 mm long, unequally cleft into five triangular lobes 4–11 mm long, outer and inner surfaces densely hispid with uniseriate trichomes, persistent on developing fruits; *corolla* white, bilabiate, tube narrowly funnelform, slightly curved near mid point, outer and inner surface glabrous, 31–34 mm long and 8–9 mm wide, upper lobes 12–15 mm long and 10–14 mm wide, lower lobe 13–20 mm long and 10–15 mm wide; *stamens* 2, ca. 7 mm long, anthers apically connate, staminodes 2; *nectary disc* cupulate, annular, deciduous from the fruit; *gynoecium* (ovary, style, and stigma) ca. 22 mm long, ovary and style glabrous, stigma applanate, bilobed; *berries* green when immature, ellipsoid, glabrous, up to 18 mm long and 11 mm wide, tipped by the basal 3 mm of the persistent style, enclosed by the persistent calyx, mature fruit not seen.

#### Distribution and ecology.

*Cyrtandra hispida* is only known from two populations in the upland rainforests of Taveuni, Fiji, where plants occur on exposed hillsides composed of volcanic cinders, and on rocky stream banks from 697–1126 m elevation (Fig. 3).

#### Phenology.

Individuals of this species were in flower when collected in July, with fruits likely becoming mature ca. 5–6 months later (December–January).

#### Etymology.

This species is named for the stiff trichomes that cover the stems, leaves, and inflorescences.

#### Phylogenetic placement.

A recent phylogenetic study by Johnson et al. (2017) placed *Cyrtandra hispida* in a weakly supported clade with four other species (*C. cephalophora* Gillespie, *C. waisaliensis* sp. nov., *C. dolichocarpa* A. Gray, *C. longifructosa* sp. nov.) that are recorded from the Fijian Islands of Viti Levu (*C. cephalophora*) and/or Vanua Levu (*C. cephalophora*, *C. waisaliensis*, *C. dolichocarpa*, *C. longifructosa*) (Fig. 4). Within this clade, *C. hispida* is most similar morphologically to *C. waisaliensis* (sp. nov., described below). Both species have large bilabiate corollas, persistent cylindrical calyces, ovate to obovate leaves, and a dense indument of long stiff trichomes covering the stems, leaves, and inflorescences. Additional sampling of species and of nuclear genic regions may be required to confidently place *C. hispida* with its closest relatives. The key provided in the taxonomic treatment by Gillett (1967) would place *C. hispida* in species Group 2 based on the branching cyme inflorescence and the persistent calyx.

#### Conservation status.

Proposed IUCN Red List Category: Endangered (EN) based on an estimated area of occupancy of < 500 km^2^ (criterion B2), known to exist at no more than five locations (B2a), projected decline in extent of occurrence (B2bi), area of occupancy (B2bii), and area, extent, and/or quality of habitat (B2biii). Although the two areas where this species has been collected are within the Taveuni Forest Reserve, the forest above Somosomo Village is currently being cleared for a hydropower dam (M. Johnson, pers. obs.). Additional threats include mining for gold and copper, invasion by plant species such as *Clidemia hirta* (L.) D. Don (Koster’s curse; M. Johnson, pers. obs), and damage from tropical cyclones. Further surveys are needed in the upland forests of Taveuni (which remain relatively unexplored, exceptions being the area surrounding Lake Tagimoucia and the road to Des Voeux Peak) to determine the extent of occurrence and population demographics of *C. hispida*.

#### Additional specimens examined.

FIJI. Taveuni: mountains above Somosomo, 16°47.67'S, 179°56.10'W, 693 m elev., 24 August 2015, *M.A. Johnson 212* (SUVA), *M.A. Johnson 215* (RSA).

#### Notes.

*Cyrtandra hispida* was observed to grow sympatrically with three species on Des Voeux Peak (*C. leucantha* A.C. Sm., *C. ciliata*, and Cyrtandra *sp.*) and three species in the mountains above Somosomo (*C. leucantha*, *C. ciliata*, *C. taviunensis* Gillespie). Several individuals were observed that appeared to be of hybrid origin in these populations, with the widespread and common *C. ciliata* inferred as one of the parents based on similar floral morphology. While the observation of ongoing hybridization in these populations suggests the possibility of *C. hispida* being of hybrid origin, none of the sympatric species have morphological characters similar to *C. hispida*. Furthermore, *C. hispida* is placed in a clade of species that are endemic to the neighboring islands of Vanua Levu and Viti Levu, and does not appear to be closely related to species endemic to Taveuni.

### Cyrtandra longifructosa

Taxon classificationPlantaeLamialesGesneriaceae

M.A.Johnson sp. nov.

urn:lsid:ipni.org:names:77174062-1

Figs 7 , 8

#### Diagnosis.

This species is closely related to *C. dolichocarpa* (Fig. 8), but differs in its glabrous elliptic-ovate leaves (vs. moderately pubescent lanceolate-ovate leaves), blades up to 22 × 9 cm (vs. blades up to 17 × 7 cm), petioles 3–9 cm long (vs. petioles 1–4 cm long), deciduous lanceolate bracts (vs. persistent ovate bracts), peduncles 2–4 mm long (vs. 5–10 mm long), pedicels 11–18 mm long (vs. 21–27 mm long), deciduous beaked calyx (vs. persistent cylindrical calyx), and corolla tube 23–29 mm long (vs. corolla tube 36–55 mm long).

#### Type.

FIJI. Vanua Levu: ca. 0.8 km NE of Waisali Village along the Waisali Creek, 16°38.51'S, 179°14.54'E, 110 m elev., 09 July 2014, *M.A. Johnson 65* with G.J. Hora (holotype: SUVA; isotype: RSA).

**Figure 7.**
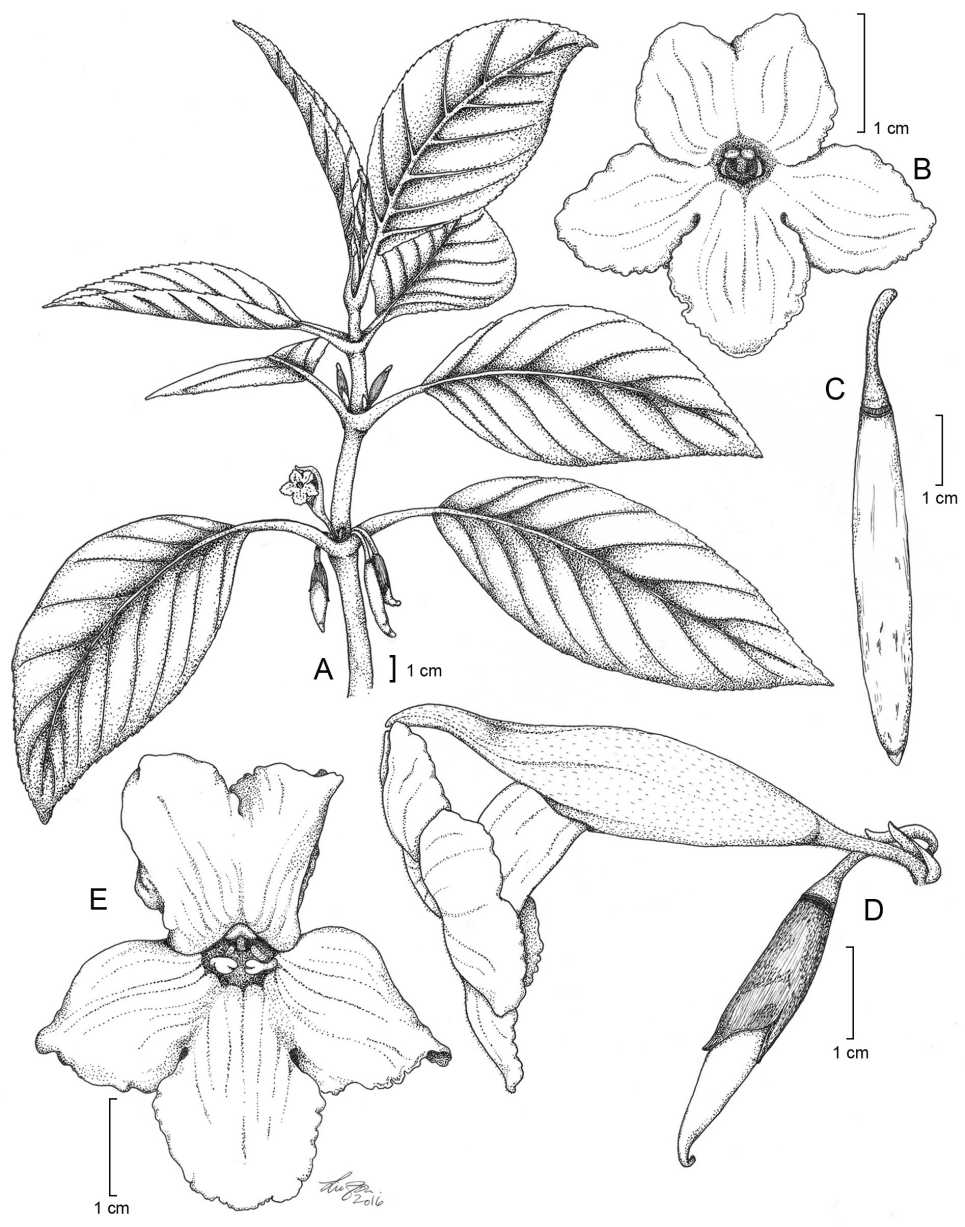
*Cyrtandra longifructosa* M.A.Johnson. **A** Habit **B** Corolla, staminate phase, anterior view **C** Mature elongate cylindrical fruit **D** Inflorescence and young fruit **E** Corolla, ovulate phase, anterior view. Drawn from Johnson 65 (SUVA, RSA), Johnson 59 (RSA), and field images.

**Figure 8.**
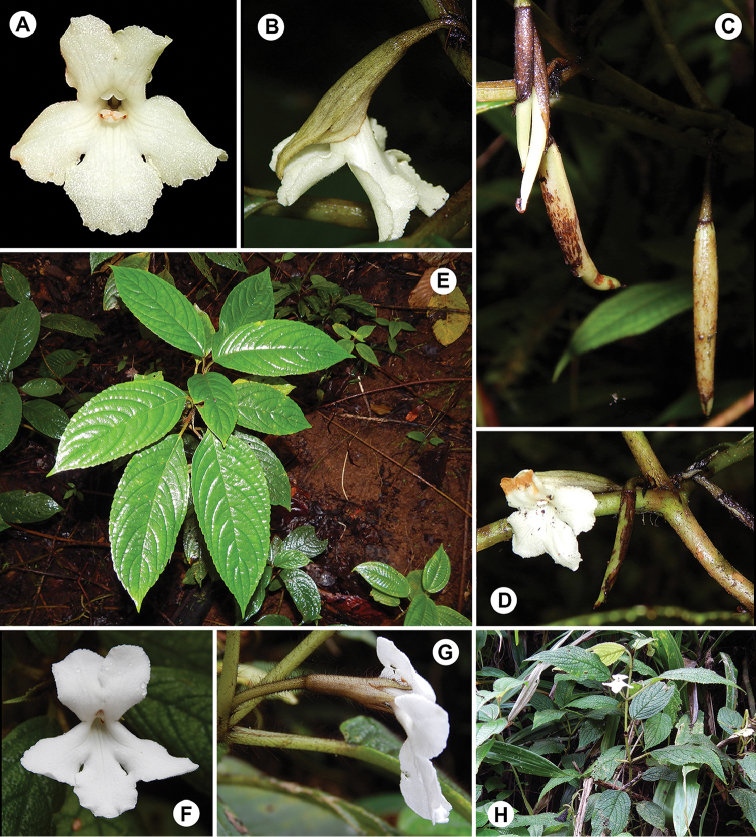
*Cyrtandra longifructosa* M.A.Johnson and closest relative (*C. dolichocarpa*) based on molecular phylogeny by Johnson et al. (2017). **A** *C. longifructosa* corolla, anterior view **B** *C. longifructosa* flower, lateral view **C** *C. longifructosa* elongate cylindrical fruits **D** *C. longifructosa* axillary inflorescence and young fruits **E** *C. longifructosa* shrub habit **F** *C. dolichocarpa* corolla, anterior view **G** *C. dolichocarpa* flower, lateral view **H** *C. dolichocarpa* shrub habit. All photos taken in the field by M. Johnson, with photos of *C. longifructosa* taken from the type collection.

#### Description.

Shrub 0.9–1.7 m tall; *stems* unbranched to few branched. *Leaves* opposite, internodes 1–5 cm long, the blades narrowly elliptic to elliptic-ovate, 17–22 cm long, 7–9 cm wide, upper and lower surface glabrous, 8–10 secondary veins on each side, these slightly impressed, margins serrulate, apex acuminate, base oblique to aequilateral and attenuate to cuneate, petioles 3–9 cm long, glabrous to glabrate; *inflorescence* an axillary cyme, 1– 2(3–4) flowers, densely pilose with black trichomes ca. 1 mm long throughout, peduncle 2–4 mm long, terminated by green bracts, 3–8 mm long, lanceolate, deciduous after anthesis, pedicels 11–18 mm long; *calyx* pale green, outer and inner surfaces pubescent with appressed uniseriate trichomes, narrowly fusiform in bud, 28–32 mm long, beaked, the 5 lobes often remaining connivent, splitting along one suture 14–19 mm long, deciduous; *corolla* white, becoming strongly bilabiate in the ovulate phase, tube cylindrical, curved near the mid point, outer surface glabrous, inner surface with uniseriate trichomes throughout and short glandular trichomes near the mouth of the tube, the tube 23–29 mm long, 5–7 mm wide, upper lobes 8–12 mm long and 8–11 mm wide, lower lobe 12–14 mm long and 11–14 mm wide; *stamens* 2, ca. 6 mm long, base of the filaments reddish orange, anthers apically connate, staminodes 3; *nectary disc* cupulate, annular, deciduous from mature fruit; *gynoecium* (ovary, style, and stigma) ca. 24 mm long, ovary glabrous, style pubescent with capitate glandular trichomes along distal ⅓ of its length, stigma bilobed; *berries* cylindrical, elongate, mature fruit to 40 mm long and 5 mm wide, glabrous, turning white at maturity.

#### Distribution and ecology.

**Figure 9.**
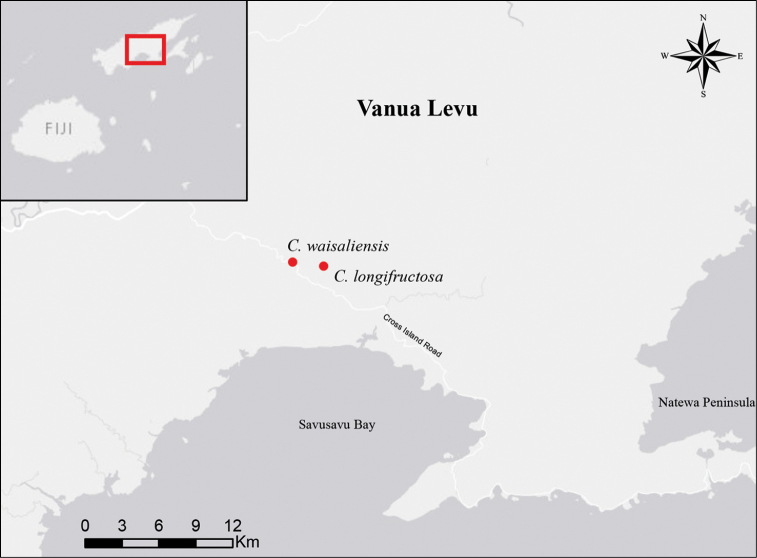
Distribution of *Cyrtandra waisaliensis* and *C. longifructosa* on Vanua Levu, Fiji.

*Cyrtandra longifructosa* is known only from one population in the Waisali region of central Vanua Levu, Fiji, where it occurs in the wet forest understory along a small creek at ca. 110 m elevation (Fig. 9).

#### Phenology.

Individuals of this species had flowers, immature fruits, and mature fruits when collected in July.

#### Etymology.

Named for the elongate cylindrical fruits, one of the diagnostic characteristics of this species.

#### Phylogenetic placement.

The phylogenetic study by Johnson et al. (2017) placed *Cyrtandra longifructosa* as sister to *C. dolichocarpa* (endemic to Vanua Levu and Rabi, Fiji) with strong support (Fig. 4). These species both have large bilabiate corollas and elongate cylindrical berries. The key provided in the taxonomic treatment by Gillett (1967) would place *C. longifructosa* in species Group 3 based on the branching cyme inflorescence, non-woody inflorescence axis, deciduous calyx, inserted anthers and styles, and calyx lobes about the same length as the calyx tube.

#### Conservation status.

Proposed IUCN Red List Category: Critically Endangered (CR): based on an estimated area of occupancy of < 10 km^2^ (criterion B2), known to exist only at a single location (B2a), projected decline in extent of occurrence (B2bi), area of occupancy (B2bii), and area, extent, and/or quality of habitat (B2biii). This species is only known from one locality in the central mountains of Vanua Levu, warranting additional surveys in areas of Vanua Levu with intact rainforest (e.g., Waisali, the Natewa Peninsula) to determine the full extent of occurrence and population demographics of *C. longifructosa*. Regions with suitable rainforest habitat on Vanua Levu are threatened by logging, mining for bauxite and gold, invasive plant species such as *Clidemia hirta* (Koster’s curse; M. Johnson, pers. obs.), and tropical cyclones.

#### Additional specimens examined.

FIJI. Vanua Levu: NE of Waisali Village along Waisali Creek, 16°38.51'S, 179°14.54'E, 110 m elev., 09 July 2014, *M.A. Johnson 59* with G.J. Hora (RSA).

#### Notes.

The population of *C. longifructosa* was observed to contain ca. 20 individuals, many of which were reproductive. No other *Cyrtandra* species were observed growing in the immediate vicinity, although the closely related species *C. dolichocarpa*, *C. waisaliensis*, and *C. cephalophora* were all collected 2.25 km W of the *C. longifructosa* population described here.

### Cyrtandra waisaliensis

Taxon classificationPlantaeLamialesGesneriaceae

M.A.Johnson sp. nov.

urn:lsid:ipni.org:names:77174063-1

Figs 10 , 11

#### Diagnosis.

The new species is closely related to *C. dolichocarpa* and *C. longifructosa* (Fig. 8), but differs in its dense bristly pubescence on the young stems, leaves, petioles, and inflorescences (vs. moderate appressed pubescence on *C. dolichocarpa*; vs. glabrous on *C. longifructosa*), cauliflorous inflorescences (vs. axillary inflorescences in both *C. dolichocarpa* and *C. longifructosa*), persistent foliaceous ovate white bracts to 10 mm long (vs. non-foliaceous green bracts to 5 mm long in *C. dolichocarpa*; vs. deciduous non-foliaceous lanceolate green bracts to 8 mm long in *C. longifructosa*), and multiple persistent foliaceous white bracteoles (vs. single deciduous non-foliaceous green bracteoles in *C. dolichocarpa*; vs. bracteoles absent in *C. longifructosa*).

**Figure 10.**
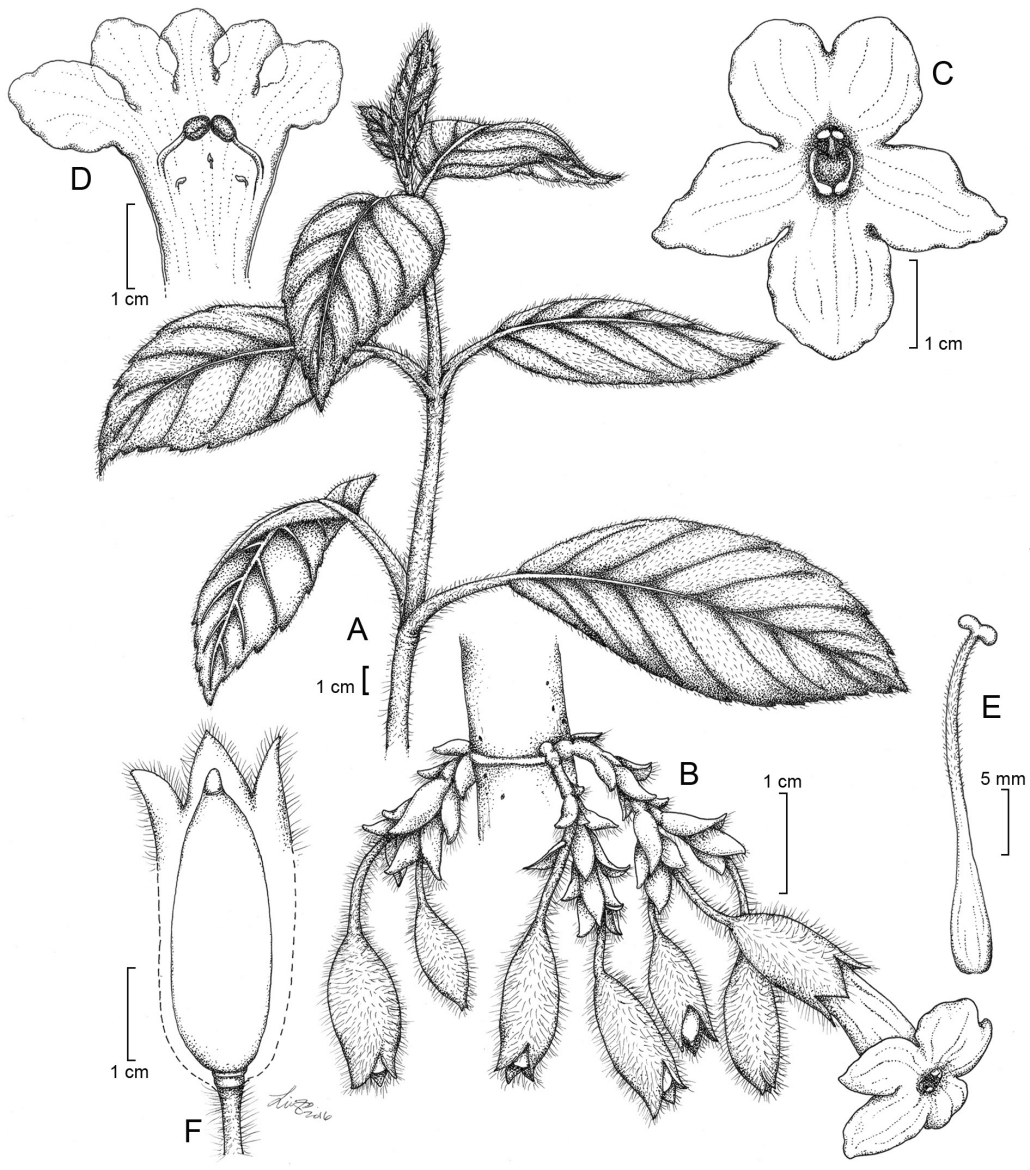
*Cyrtandra waisaliensis* M.A.Johnson. **A** Habit **B** Cauliflorous inflorescence **C** Corolla, anterior view **D** Corolla, longitudinal section **E** Gynoecium **F** Calyx, longitudinal section and young fruit. Drawn from Johnson 48 (RSA), Johnson 50 (SUVA, RSA), and field images.

**Figure 11.**
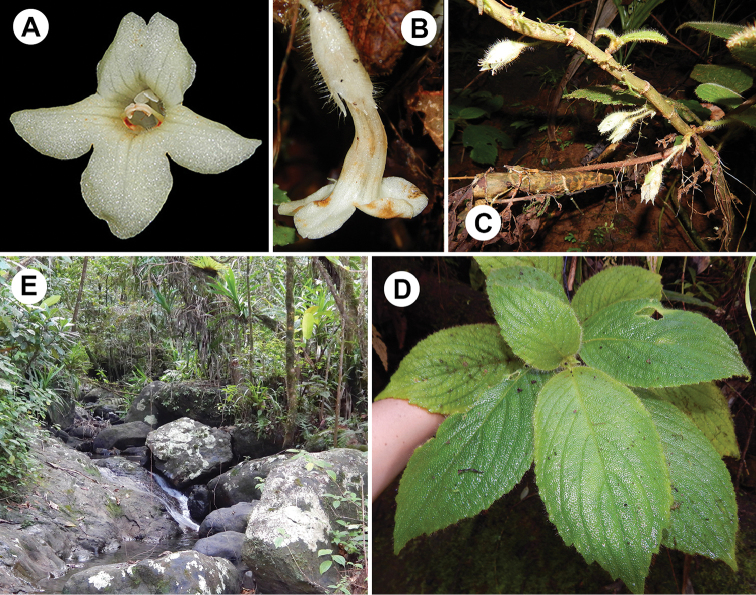
*Cyrtandra waisaliensis* M.A.Johnson. **A** Corolla, anterior view **B** Flower, lateral view **C** Cauliflorous cyme inflorescence **D** Shrub habit **E** Rainforest understory habitat. All photos from the type collection, taken in the field by M. Johnson.

#### Type.

FIJI. Vanua Levu: Waisali Forest Reserve along the lower portion of the Savuqoro Creek, 16°38.39'S, 179°13.25'E, 338 m elev., 07 July 2014, *M.A. Johnson 50* with G.J. Hora (holotype: SUVA; isotype: RSA).

#### Description.

Shrub 0.7–2.6 m tall; *stems* few- to many-branched, young stems covered in hirsute trichomes. *Leaves* opposite, those at a node unequal, internodes 2–8 cm long, blades ovate to obovate, 13–24 cm long, 6–12 cm wide, upper and lower surfaces densely hirsute with uniseriate multicellular trichomes, 6–7 secondary veins on each side, margins serrate, apex acuminate, base oblique to aequilateral and attenuate to cuneate, petioles 3–11 cm long, densely hirsute, trichomes ca. 5 mm long; *inflorescence* of cauliflorous cymes, 2–8 flowered, cymules 1–4 flowered, densely hirsute throughout, peduncle 3–8 mm long, terminated by persistent foliaceous white bracts, ovate to lanceolate, 3–10 mm long, outer surface glabrous to glabrate, inner surface densely hirsute, pedicels 21–40 mm long, subtended by multiple persistent bracteoles similar to and closely subtended by the outer bracts; *calyx* white, cylindrical, densely hirsute, 23–37 mm long, unequally cleft into 5 triangular lobes, 4–14 mm long, persistent; *corolla* white, bilabiate, tube funnelform, curved near mid point, outer surface glabrous to glabrate, inner surface with capitate glandular trichomes near the mouth of the tube, 23–27 mm long, 6–9 mm wide, upper lobes 11–12 mm long and 8–11 mm wide, lower lobe 13–17 mm long and 10–15 mm wide; *stamens* 2, 3–6 mm long, base of the filaments reddish orange, anthers apically connate, staminodes 3; *nectary disc* prominent, annular, 2 mm high; *gynoecium* (ovary, style, and stigma) 16–26 mm long, ovary glabrous, style pubescent with capitate glandular trichomes along distal ⅓ of length, stigma bilobed; *berries* ellipsoid, green when immature, enclosed by the persistent calyx, mature fruit not seen.

#### Distribution and ecology.

*Cyrtandra waisaliensis* is known only from one population in the Waisali Forest Reserve on Vanua Levu, Fiji at 300–360 m elevation, occurring in the dense forest understory alongside a creek (Fig. 9).

#### Phenology.

Flowers and immature fruits were observed in July, with fruits likely becoming mature ca. 5–6 months later (December–January).

#### Etymology.

The new species is named after the area of Vanua Levu where it was collected, Waisali Forest Reserve.

#### Phylogenetic placement.

A recent phylogenetic study by Johnson et al. (2017) supported the placement of *Cyrtandra waisaliensis* as sister to *C. longifructosa* (endemic to Vanua Levu) and *C. dolichocarpa* (endemic to Vanua Levu and Rabi; Fig. 4). These species all have large bilabiate corollas, and both *C. dolichocarpa* and *C. longifructosa* have elongate cylindrical white fruits. However, *C. waisaliensis* is also morphologically similar to *C. hispida*; these species share bilabiate corollas, persistent cylindrical calyces, and a dense indument of stiff uniseriate trichomes. *Cyrtandra hispida* is currently placed in a polytomy with *C. cephalophora* and the clade comprising *C. waisaliensis*, *C. longifructosa*, and *C. dolichocarpa*. The key provided in the taxonomic treatment by Gillett (1967) would place *C. waisaliensis* in species Group 2, based on the branching cyme inflorescence and the persistent calyx.

#### Conservation status.

Proposed IUCN Red List Category: Critically Endangered (CR) based on an estimated area of occupancy of < 10 km^2^ (criterion B2), known to exist at only a single location (B2a), projected decline in extent of occurrence (B2bi), area of occupancy (B2bii), and area, extent, and/or quality of habitat (B2biii). This species is only known from one locality in the central mountains of Vanua Levu, warranting additional surveys in areas of Vanua Levu with intact rainforest (e.g., Waisali, the Natewa Peninsula) to determine the full extent of occurrence and population demographics of *C. waisaliensis*. Regions with suitable rainforest habitat on Vanua Levu are threatened by logging, mining for bauxite and gold, invasive plant species such as *Clidemia hirta* (Koster’s curse; M. Johnson, pers. obs.), and tropical cyclones.

#### Additional specimens examined.

FIJI. Vanua Levu: Waisali Forest Reserve along the lower portion of the Savuqoro Creek, 16°38.34'S, 179°13.18'E, 355 m elev., 07 July 2014, *M.A. Johnson 48* with G.J. Hora (RSA).

#### Notes.

The observed population of *C. waisaliensis* was comprised of ca. 20 individuals, many of which were reproductive. A single individual appeared to be of hybrid origin, with the putative parents being *C. waisaliensis* and *C. cephalophora* based on morphological characters intermediate between these two species.

## Conclusions

The recognition of *C. gregoryi*, *C. hispida*, *C. longifructosa*, and *C. waisaliensis* brings the new total of Fijian *Cyrtandra* species to 41. The four new species of *Cyrtandra* described here demonstrate that the islands of Fiji remain poorly explored botanically, at least in some regions. Of the 28 Fijian *Cyrtandra* species collected during field expeditions in 2014 and 2015, four of these were new to science. An additional four *Cyrtandra* species could not be keyed out to any of the existing species due to a lack of reproductive material. However, with further field study it is possible that these, along with other future collections, may be identified as new species.

## Supplementary Material

XML Treatment for Cyrtandra gregoryi

XML Treatment for Cyrtandra hispida

XML Treatment for Cyrtandra longifructosa

XML Treatment for Cyrtandra waisaliensis
